# An Analysis of Cytomegalovirus-Specific Cell-Mediated Immunity in a Phase 3, Randomized, Placebo-Controlled Trial of Letermovir Prophylaxis in Cytomegalovirus-Seropositive Recipients of an Allogeneic Hematopoietic Cell Transplant

**DOI:** 10.1093/cid/ciaf646

**Published:** 2025-12-01

**Authors:** Genovefa A Papanicolaou, Sanjeet S Dadwal, Ella J Ariza-Heredia, Patrizia Chiusolo, Michele I Morris, Eleni Tholouli, Barbara Haber, Natalya Broyde, Christopher L Gilbert, Cyrus Badshah

**Affiliations:** Division of Infectious Diseases, Memorial Sloan Kettering Cancer Center, New York, New York, USA; Weill Cornell Medical College, Cornell University, New York, New York, USA; Division of Infectious Diseases, Department of Medicine, City of Hope National Medical Center, Duarte, California, USA; Department of Infectious Diseases, Infection Control, and Employee Health, Division of Internal Medicine, University of Texas MD Anderson Cancer Center, Houston, Texas, USA; Hematology and Stem Cell Transplantation Unit, Fondazione Policlinico Universitario A. Gemelli IRCCS, Roma, Italy; Division of Infectious Diseases, University of Miami Miller School of Medicine, Miami, Florida, USA; Department of Haematology, Manchester Royal Infirmary, Manchester, United Kingdom; Merck & Co., Inc., Rahway, New Jersey, USA; Merck & Co., Inc., Rahway, New Jersey, USA; Merck & Co., Inc., Rahway, New Jersey, USA; Merck & Co., Inc., Rahway, New Jersey, USA

**Keywords:** cytomegalovirus-specific cell-mediated immunity, hematopoietic cell transplant, interferon γ release assays, letermovir, QuantiFERON-CMV assay

## Abstract

**Background:**

Cytomegalovirus (CMV)-specific cell-mediated immunity (CMV-CMI) prevents CMV reactivation after hematopoietic cell transplant (HCT). Trends in CMV-CMI using the QuantiFERON-CMV assay and its clinical utility in a Phase 3 study were evaluated.

**Methods:**

Cytomegalovirus-seropositive allogeneic HCT recipients who received letermovir prophylaxis through ∼100 days post-HCT were randomized to receive letermovir or placebo through ∼200 days post-HCT. QuantiFERON-CMV was performed at 100 days, 200 days, and Week 48 post-HCT. QuantiFERON-CMV results after prophylaxis completion (∼100 and ∼200 days post-HCT in placebo and letermovir groups, respectively) were evaluated for predicting postprophylaxis clinically significant CMV infection (CS-CMVi) through Week 48 post-HCT.

**Results:**

QuantiFERON-CMV assay positivity was similar at study entry between letermovir (n = 82) and placebo (n = 47) groups (30.5% vs 31.9%), higher for placebo group at 200 days post-HCT (48.8% vs 74.5%), and comparable between groups at Week 48 post-HCT (65.9% vs 74.5%). Postprophylaxis CS-CMVi occurred by Week 48 in 10.5% (6/57) of participants with a positive result versus 19.0% (8/42) with a negative result at 200 days post-HCT in the letermovir group and 10.3% (3/29) of participants with a positive result versus 20.7% (6/29) with a negative result at 100 days post-HCT in the placebo group. Assay sensitivity, specificity, positive predictive value, and negative predictive value were 60.0%, 57.1%, 89.5%, and 19.0% for tests at 200 days post-HCT (letermovir) and 53.1%, 66.7%, 89.7%, and 20.7% for tests at 100 days post-HCT (placebo).

**Conclusions:**

Positive QuantiFERON-CMV results increased after letermovir prophylaxis completion. Predictive utility of the QuantiFERON-CMV assay may be limited in the HCT setting.

## Teaser

In this analysis of samples from a large, Phase 3, randomized, global study, the clinical utility of the QuantiFERON-CMV assay for predicting protection against clinically significant cytomegalovirus infection was limited in the hematopoietic cell transplant setting.

## Graphical Abstract

**Graphical Abstract:**
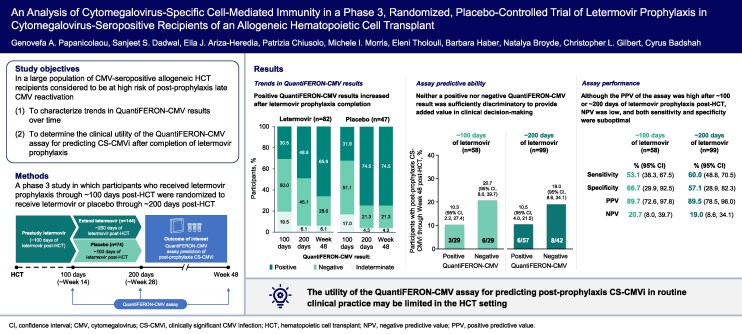

**(See the Editorial Commentary by Natori and Sester on pages e325–7.)**

Recipients of allogeneic hematopoietic cell transplant (HCT) who are seropositive for cytomegalovirus (CMV) are at high risk of CMV reactivation and infection, which is associated with considerable morbidity and mortality [1–3]. The risk of CMV reactivation in this population is highest within the first 100 days (∼14 weeks) after transplantation and decreases as the immune system reconstitutes [4]. However, some recipients of HCT remain at high risk of CMV infection and disease beyond 100 days post-HCT, possibly due to delayed immune reconstitution, continued immunosuppression to treat graft-versus-host disease, or new-onset graft-versus-host disease after immunosuppression is tapered [5–7].

Letermovir is a CMV terminase complex inhibitor approved for prophylaxis of CMV infection and disease in recipients of allogeneic HCT who are seropositive for CMV [8, 9]. A pivotal Phase 3 registrational study demonstrated the safety and efficacy of letermovir administered through ∼100 days post-HCT for prevention of clinically significant CMV infection (CS-CMVi) [10], leading to its approval in 2017 [8]. However, after letermovir prophylaxis was completed, a ∼12% increased incidence of CS-CMVi was observed between 100 and 200 days post-HCT [11].

On the basis of these results, the subsequent Phase 3, multicenter, randomized, double-blind, placebo-controlled, extended-duration prophylaxis study was conducted to evaluate the safety and efficacy of extending the duration of letermovir prophylaxis to ∼200 days (∼28 weeks) post-HCT in CMV-seropositive recipients of allogeneic HCT who were considered to be at high risk of developing late CS-CMVi [11]. Participants who had received ∼100 days of letermovir post-HCT were randomized to receive letermovir or placebo through 200 days post-HCT. Thus, participants received ∼200 or ∼100 days of letermovir in the letermovir and placebo groups, respectively. Extending letermovir prophylaxis to ∼200 days post-HCT reduced the incidence of late CS-CMVi from Week 14 to Week 28 post-HCT in this high-risk population [letermovir, 1.4% {2/144}; placebo, 17.6% {13/74}]. However, after completion of extended-duration letermovir prophylaxis, the incidence of CS-CMVi increased to 13.2% (19/144) in the letermovir group versus 18.9% (14/74) in the placebo group through Week 38 post-HCT, and these levels remained stable at Week 48 post-HCT. These results suggest that even further extension of letermovir prophylaxis may benefit some patients. The decision to extend letermovir prophylaxis should weigh the risks and benefits of continued virologic suppression and consider the patient's immune reconstitution. Therefore, a standardized and accessible test to assess immune reconstitution is of considerable clinical value.

Cytomegalovirus-specific cell-mediated immunity (CMV-CMI) is important in controlling CMV reactivation after allogeneic HCT [12]. Antigen exposure during active CMV replication may enhance the development of CMV-CMI [13, 14]. Letermovir targets a later stage of CMV replication as compared with DNA polymerase inhibitors [14, 15]. Recent findings suggest that letermovir prophylaxis delays CMV-specific cellular reconstitution compared with naturally occurring infection [14].

There has been an increasing interest in monitoring CMV-CMI using interferon γ (IFN-γ) release assays (IGRAs) to better risk stratify immunocompromised patients and guide decisions on extending prophylactic therapy [16–21]. These assays assess CMV-CMI by measuring IFN-γ released by CD4+ or CD8+ T cells in the presence of CMV antigens [16, 17]. However, many assays for CMV-CMI lack standardization and require laboratory expertise and specialized technology, which may not be widely available [16, 22]. The QuantiFERON-CMV (Qiagen, Inc; Hilden, Germany) assay is a commercially available IGRA for assessment of CMV-CMI [22–24]. It measures CD8+ T-cell IFN-γ responses to epitopes of several CMV proteins including pp65, pp50, the glycoprotein gB, and the immediate early IE-1 antigen [24, 25]. The QuantiFERON-CMV assay offers advantages over alternative approaches, including validated cutoff values, short turnaround time, and the ability to use whole blood without separation of peripheral blood lymphocytes [22, 24, 25]. Its limitations are that it does not quantify CMV-specific cell numbers or assess other cells, particularly CMV-specific CD4+ T cells, which are critical to CMV-CMI [24, 26].

The objectives of this study were to (1) characterize trends in QuantiFERON-CMV results as a potential indicator of CMV-CMI through Week 48 post-HCT and (2) determine the clinical utility of this assay for predicting CS-CMVi after completion of letermovir prophylaxis using samples from the extended-duration prophylaxis study.

## METHODS

### Study Design and Participants

The extended-duration prophylaxis study (NCT03930615) was a global, Phase 3, multicenter, randomized, double-blind, placebo-controlled study. The study design and eligibility criteria have been previously described [11]. Adult CMV-seropositive recipients of allogeneic HCT who received letermovir prophylaxis through 100 days post-HCT (∼14 weeks post-HCT) and were considered to be at high risk of postprophylaxis late CMV reactivation using prespecified criteria (defined in Supplementary Methods) were randomized to either extend letermovir prophylaxis or receive placebo for an additional 100 days (∼28 weeks post-HCT). Therefore, participants in the letermovir group received ∼200 days of letermovir post-HCT, whereas those in the placebo group received ∼100 days of letermovir post-HCT.

Investigators monitored participants for the development of CS-CMVi through 48 weeks post-HCT, defined as CMV infection requiring preemptive therapy for viremia or onset of end-organ disease. This definition of CS-CMVi aligns with current consensus definitions [27].

The extended-duration prophylaxis study was conducted in accordance with the principles of Good Clinical Practice and was approved by the appropriate institutional review boards and regulatory agencies. All participants provided informed consent.

### Assessment of CMV-CMI Using QuantiFERON-CMV Assay

**Figure 1.**
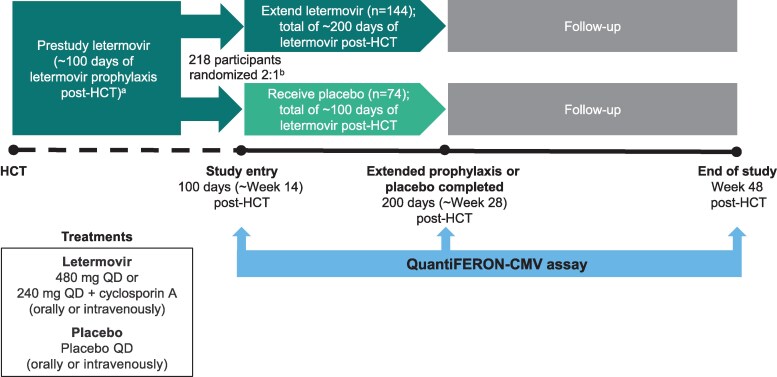
Study design. Abbreviations: CMV, cytomegalovirus; HCT, hematopoietic cell transplant; QD, once daily. ^a^Participants initiated letermovir within 28 d post-HCT and continued through 100 d post-HCT. ^b^Stratified by study center and haploidentical donor (yes or no).

Figure 1 shows the study design. Whole blood samples were prospectively collected at prespecified time points: 100 days, 200 days, and Week 48 (study completion) post-HCT. The QuantiFERON-CMV assay was selected for its commercial availability and logistical feasibility in a global multicenter study. Preliminary incubation was conducted at each site, and analysis was performed in batches at a central laboratory in accordance with manufacturer recommendations by personnel who were blinded to clinical outcomes. Assay results reported as being reactive, nonreactive, and indeterminate were interpreted as being positive, negative, and indeterminate for CMV-CMI, respectively (Supplementary Methods). Details of the assay can be found in the package insert (https://www.quantiferon.com/wp-content/uploads/2018/10/L1075110-R05-QF-CMV-ELISA-IFU-CE.pdf) [23].

### Outcome of Interest

This prespecified analysis evaluated the clinical utility of the QuantiFERON-CMV assay in predicting the incidence of postprophylaxis CS-CMVi through Week 48 post-HCT.

### Analysis Populations

To characterize trends in QuantiFERON-CMV results, the proportion of participants with positive, negative, or indeterminate results available in the letermovir and placebo groups were assessed at 100 days, 200 days, and Week 48 (study completion) post-HCT. This analysis population included all randomly assigned participants who received ≥1 dose of study treatment with QuantiFERON-CMV results available at all 3 time points.

In a separate analysis population, the clinical utility of the QuantiFERON-CMV assay was evaluated by correlating the proportion of participants with a positive or negative QuantiFERON-CMV result after completion of prophylaxis with postprophylaxis CS-CMVi through Week 48 post-HCT. Analyses were conducted in the following:

1. Participants with a positive or negative result at 100 days post-HCT in the placebo group (at study entry when ∼100 days of letermovir prophylaxis was completed). Participants with indeterminate and negative results were pooled in a separate analysis.
2. Participants with a positive or negative result at 200 days post-HCT in the letermovir group [when ∼200 days of prophylaxis was completed; the median duration of letermovir prophylaxis in this group was 98 days {range, 39–104 days} after study entry]. Participants with indeterminate and negative results were pooled in a separate analysis.
3. A pooled analysis of participants with a positive or negative result at 100 or 200 days post-HCT in the placebo and letermovir groups.

### Statistical Analysis

Descriptive statistics were used to summarize baseline demographics and characteristics of participants.

Sensitivity, specificity, positive predictive value (PPV), and negative predictive value (NPV) of the assay were calculated to determine if QuantiFERON-CMV results after prophylaxis completion (100 or 200 days post-HCT in the placebo and letermovir groups, respectively) could be predictive of protection against CS-CMVi through Week 48 post-HCT. In these analyses, sensitivity expressed the likelihood of whether the QuantiFERON-CMV assay could correctly identify if a participant with a positive result would not develop postprophylaxis CS-CMVi (ie, correctly identify participants who were protected). Specificity measured whether a negative result correctly identified if a participant would develop postprophylaxis CS-CMVi. Positive predictive value indicated the probability of not experiencing postprophylaxis CS-CMVi with a positive QuantiFERON-CMV result, whereas NPV indicated the probability of developing postprophylaxis CS-CMVi with a negative QuantiFERON-CMV result. To further evaluate the clinical utility of the QuantiFERON-CMV assay, participants with negative and indeterminate QuantiFERON-CMV results were pooled.

A univariate analysis was conducted to identify whether baseline factors correlated with QuantiFERON-CMV results at Week 48 post-HCT. Factors identified in the univariate analysis were included in a subsequent stepwise regression model. Nonsignificant factors were removed using a 0.05 level for significance.

## RESULTS

### Analysis Populations

Study disposition and baseline demographics of the full analysis set have been previously published [11]. Supplementary Figure 1 shows participant disposition for the analysis population used to assess trends in QuantiFERON-CMV results (participants with results available at all 3 time points). This population included 82 and 47 participants in the letermovir and placebo groups, respectively. Baseline characteristics were comparable with those of the overall study population [11] and between the 2 treatment groups (Table 1). Most participants were male and White, and the mean age was 53.2 years. More than half received T-cell–depleting induction therapy; antithymocyte globulin and alemtuzumab use were similar between groups (Table 1).

**Table 1.** Demographics and Baseline Characteristics of Participants in the Analysis Population for Assessing Trends in QuantiFERON-CMV Results^a^

	Letermovir (n = 82)^b^	Placebo (n = 47)^c^	Total (N = 129)
Sex, male, n (%)	58 (70.7)	25 (53.2)	83 (64.3)
Age, mean (SD), years	53.6 (13.3)	52.6 (14.1)	53.2 (13.5)
Race, n (%)			
Asian	13 (15.9)	4 (8.5)	17 (13.2)
Black or African American	1 (1.2)	0	1 (0.8)
Multiple	1 (1.2)	1 (2.1)	2 (1.6)
Native Hawaiian or other Pacific Islander	1 (1.2)	0	1 (0.8)
White	61 (74.4)	38 (80.9)	99 (76.7)
Not available^d^	5 (6.1)	4 (8.5)	9 (7.0)
Donor type, n (%)			
Matched related	8 (9.8)	6 (12.8)	14 (10.9)
Mismatched related	28 (34.1)	17 (36.2)	45 (34.9)
Matched unrelated	23 (28.0)	14 (29.8)	37 (28.7)
Mismatched unrelated	23 (28.0)	10 (21.3)	33 (25.6)
Donor CMV serostatus, n (%)			
Positive	46 (56.1)	34 (72.3)	80 (62.0)
Negative	36 (43.9)	13 (27.7)	49 (38.0)
Stem cell source, n (%)			
Peripheral blood	69 (84.1)	36 (76.6)	105 (81.4)
Bone marrow	6 (7.3)	6 (12.8)	12 (9.3)
Cord blood	7 (8.5)	5 (10.6)	12 (9.3)
T-cell–depleting induction therapy, n (%)	45 (54.0)	27 (57.4)	72 (55.8)
Ex vivo T-cell–depleted grafts	6 (7.3)	4 (8.5)	10 (7.8)
Antithymocyte globulin	38 (46.3)	23 (48.9)	61 (47.3)
Alemtuzumab	6 (7.3)	4 (8.5)	10 (7.8)
Conditioning regimen use, n (%)			
Myeloablative	38 (46.3)	19 (40.4)	57 (44.2)
Reduced intensity conditioning	30 (36.6)	18 (38.3)	48 (37.2)
Nonmyeloablative	14 (17.1)	10 (21.3)	24 (18.6)

Abbreviations: CMV, cytomegalovirus; HCT, hematopoietic cell transplant.

^a^Includes all participants who had QuantiFERON-CMV results at the 100 d post-HCT, 200 d post-HCT, and Week 48 post-HCT time points.

^b^Participants received ∼100 d of letermovir prophylaxis post-HCT and then completed extended-duration letermovir prophylaxis through 200 d post-HCT.

^c^Participants completed ∼100 d of letermovir prophylaxis post-HCT and then received placebo through 200 d post-HCT.

^d^Participants did not know their race or chose not to report due to local regulations.

Participant disposition for the analysis population used to assess the clinical utility of the QuantiFERON-CMV assay is presented in Supplementary Figure 2. This population included participants with a positive or negative assay result available at 200 days post-HCT in the letermovir group (n = 99) and at 100 days post-HCT in the placebo group (n = 58). Baseline characteristics were comparable with those of the overall study population [11] and between the 2 treatment groups (Supplementary Table 1).

### Trends in QuantiFERON-CMV Results

**Figure 2.**
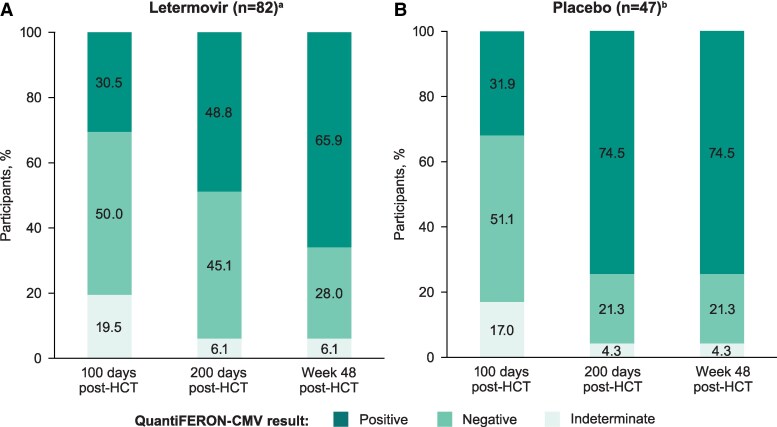
Characterization of trends in QuantiFERON-CMV results through Week 48 post-HCT in the analysis population for assessing trends in QuantiFERON-CMV results in the (*A*) letermovir and (*B*) placebo groups. The analysis population includes all participants who had QuantiFERON-CMV results at the 100 d post-HCT, 200 d post-HCT, and Week 48 post-HCT time points. Abbreviations: CMV, cytomegalovirus; HCT, hematopoietic cell transplant. ^a^Participants received ∼100 d of letermovir prophylaxis post-HCT and then completed extended-duration letermovir prophylaxis through 200 d post-HCT. ^b^Participants completed ∼100 d of letermovir prophylaxis post-HCT then received placebo through 200 d post-HCT.

At study entry, when all participants had received ∼100 days of letermovir prophylaxis, the percentage of participants who had positive QuantiFERON-CMV results (indicating CMV-CMI) was comparable between treatment groups (letermovir, 30.5%; placebo, 31.9%; Figure 2). Thereafter, the percentage of participants with a positive QuantiFERON-CMV result increased in both treatment groups through 200 days post-HCT but was lower in the letermovir group (48.8%), where participants received letermovir up to this time point, compared with the placebo group (74.5%), where letermovir prophylaxis was completed at 100 days post-HCT. In the letermovir group, the percentage of participants with positive results continued to rise after completion of ∼200 days of prophylaxis such that by Week 48 post-HCT, the rates of positive results were similar across treatment groups (letermovir, 65.9%; placebo, 74.5%).

### Baseline Factors and QuantiFERON-CMV Results at Week 48 Post-HCT

Baseline factors including conditioning regimen, stem cell source, donor type, and donor CMV serostatus were not found to be meaningfully associated with QuantiFERON-CMV results at Week 48 post-HCT (see Supplementary Results).

### Clinical Utility of QuantiFERON-CMV Assay

Among the 58 participants in the placebo group who received ∼100 days of letermovir post-HCT, assay sensitivity and specificity for developing postprophylaxis CS-CMVi through Week 48 post-HCT were 53.1% and 66.7%, respectively, and the PPV and NPV were 89.7% and 20.7%, respectively (Table 2). Similar results were obtained among the 99 participants in the letermovir group who received ∼200 days of letermovir post-HCT (Table 2). Assay performance was comparable when indeterminate and negative results were pooled (Supplementary Table 2).

**Table 2.** QuantiFERON-CMV Assay Performance for Predicting Postprophylaxis CS-CMVi Through Week 48 in the Analysis Population for Evaluating the Clinical Utility of QuantiFERON-CMV Assay

Assay Performance for Placebo Group Based on Results After ∼100 d of Letermovir Prophylaxis^a^			
QuantiFERON-CMV Result	Did Not Develop Postprophylaxis CS-CMVi (n = 49)	Developed Postprophylaxis CS-CMVi (n = 9)	PPV/NPV, % (95% CI)
Positive (n = 29)	26 (TP)	3 (FP)	PPV, 89.7 (72.6, 97.8)
Negative (n = 29)	23 (FN)	6 (TN)	NPV, 20.7 (8.0, 39.7)
Sensitivity/specificity, % (95% CI)	Sensitivity, 53.1 (38.3, 67.5)	Specificity, 66.7 (29.9, 92.5)	…

Assay Performance for Letermovir Group Based on Results After ∼200 d of Letermovir Prophylaxis^b^			
QuantiFERON-CMV Result	Did Not Develop Postprophylaxis CS-CMVi (n = 85)	Developed Postprophylaxis CS-CMVi (n = 14)	PPV/NPV, % (95% CI)
Positive (n = 57)	51 (TP)	6 (FP)	PPV, 89.5 (78.5, 96.0)
Negative (n = 42)	34 (FN)	8 (TN)	NPV, 19.0 (8.6, 34.1)
Sensitivity/specificity, % (95% CI)	Sensitivity, 60.0 (48.8, 70.5)	Specificity, 57.1 (28.9, 82.3)	…

The 95% CIs were calculated based on exact binomial tests. Sensitivity = $\frac{TP}{TP+FN}$; specificity = $\frac{TN}{TN+FP}$; PPV = $\frac{TP}{TP+FP}$; and NPV = $\frac{TN}{TN+FN}$.

Abbreviations: CI, confidence interval; CMV, cytomegalovirus; CS-CMVi, clinically significant CMV infection; FN, false negative; FP, false positive; HCT, hematopoietic cell transplant; NPV, negative predictive value; PPV, positive predictive value; TN, true negative; TP, true positive.

^a^Samples for QuantiFERON-CMV assay collected after completion of ∼100 d of letermovir prophylaxis. Analysis population includes all placebo group participants who had positive or negative QuantiFERON-CMV results at 100 d post-HCT.

^b^Samples for QuantiFERON-CMV assay collected after completion of ∼200 d of letermovir prophylaxis. Analysis population includes all letermovir group participants who had positive or negative QuantiFERON-CMV results at 200 d post-HCT.

**Figure 3.**
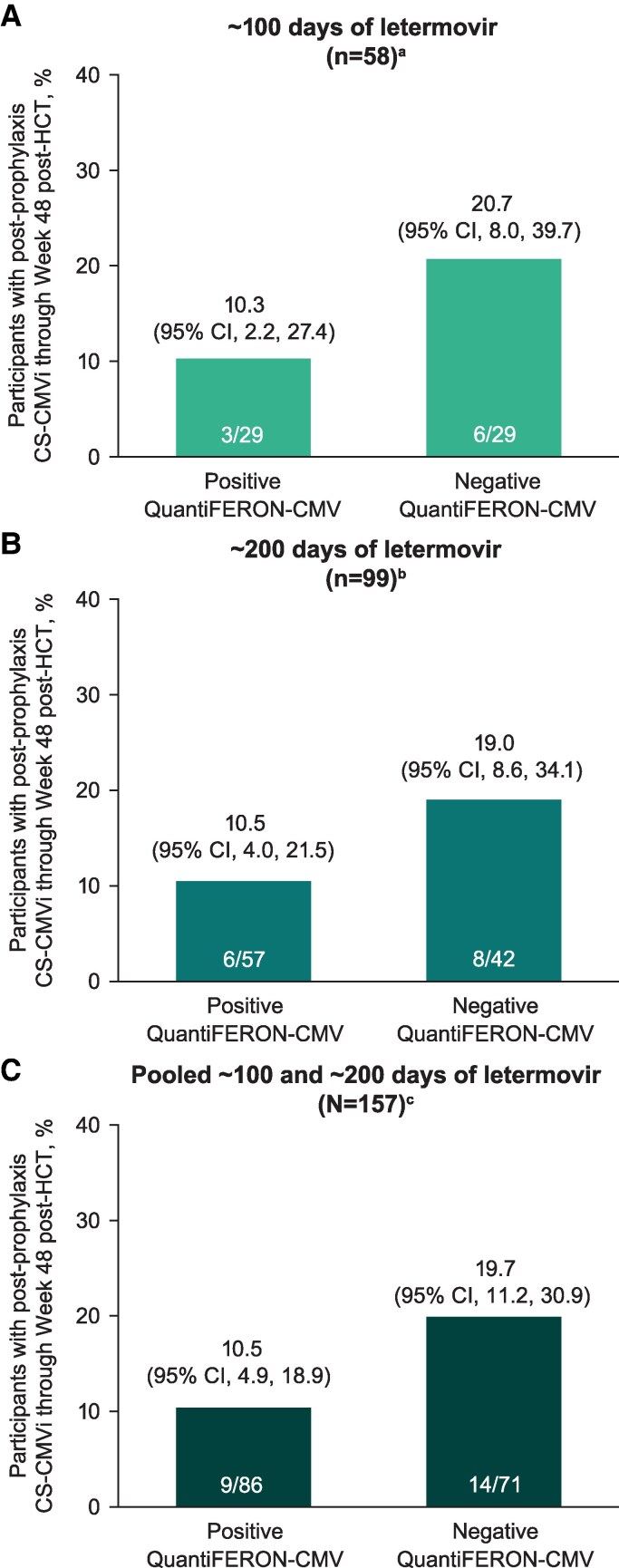
Characterization of clinical utility of QuantiFERON-CMV assay for predicting postprophylaxis CS-CMVi through Week 48 post-HCT in the analysis population for evaluating the clinical utility of QuantiFERON-CMV assay. QuantiFERON-CMV results were evaluated after completion of letermovir prophylaxis at the (*A*) 100 d post-HCT time point in the placebo group, (*B*) 200 d post-HCT time point in the letermovir group, and (*C*) 100 d and 200 d post-HCT time points in the placebo and letermovir groups, respectively, in a pooled analysis. Abbreviations: CI, confidence interval; CMV, cytomegalovirus; CS-CMVi, clinically significant CMV infection; HCT, hematopoietic cell transplant. ^a^Analysis population includes all participants who had QuantiFERON-CMV results at 100 d post-HCT in the placebo group. ^b^Analysis population includes all participants who had QuantiFERON-CMV results at 200 d post-HCT in the letermovir group. ^c^Analysis population includes all participants who had QuantiFERON-CMV results at 100 d post-HCT in the placebo group and 200 d post-HCT in the letermovir group.

The predictive ability of a positive or negative QuantiFERON-CMV result for postprophylaxis CS-CMVi through Week 48 post-HCT was assessed after completion of ∼100 days (Figure 3*A*) or ∼200 days (Figure 3*B*) of letermovir and in a pooled analysis of participants who completed either ∼100 or ∼200 days of letermovir (Figure 3*C*). Regardless of prophylaxis duration, a positive result after prophylaxis completion was associated with a ∼10% incidence of postprophylaxis CS-CMVi through Week 48 post-HCT, similar to the 13.2% incidence rate through Week 48 after ∼200 days of prophylaxis in the letermovir group of the extended-duration prophylaxis study [11]. Likewise, a negative result after prophylaxis completion was associated with a ∼20% incidence of postprophylaxis CS-CMVi, similar to the 18.9% incidence rate through Week 48 after ∼100 days of letermovir prophylaxis in the placebo group of the study, regardless of prophylaxis duration [11]. Thus, the assay was not useful for guiding timing of prophylaxis discontinuation, nor did it provide added value over using prophylaxis for a preset duration followed by surveillance without QuantiFERON-CMV testing.

## DISCUSSION

Cytomegalovirus infection remains an important cause of morbidity and mortality in recipients of allogeneic HCT [1–3]. In the clinical setting, monitoring trends in CMV-CMI using a standardized and accessible assay may be useful for identifying patients at risk of postprophylaxis CS-CMVi. Here, we evaluated trends in CMV-CMI using the QuantiFERON-CMV assay and the assay's utility for predicting CS-CMVi after completion of letermovir prophylaxis. The samples for the assay were collected in a prospective fashion at prespecified time points during a large, global, double-blind, multicenter, Phase 3 study evaluating the extension of letermovir prophylaxis in recipients of allogeneic HCT who were seropositive for CMV [11].

At study entry, when all participants had received letermovir through ∼100 days post-HCT, approximately one-third of participants had a positive QuantiFERON-CMV result indicating CMV-CMI. Over the next 100 days, the percentage of participants with a positive QuantiFERON-CMV result increased across both treatment groups but was lower in the letermovir (48.8%) group compared with the placebo (74.5%) group, where letermovir was completed ∼100 days earlier. Thus, we observed delayed QuantiFERON-CMV positivity in the letermovir group, possibly due to longer duration of virologic suppression; at this time point, the CS-CMVi rate was 1.4% in the letermovir group compared with 17.6% in the placebo group in the extended-duration prophylaxis study [11].

By Week 48 post-HCT, assay positivity rates were comparable between groups (letermovir, 65.9%; placebo, 74.5%); at this time point, CS-CMVi had rebounded to 13.2% in the letermovir group compared with 18.9% in the placebo group [11]. This is consistent with studies finding that a low level of viral replication is necessary for the optimal development of CMV-CMI after allogeneic HCT [13, 14].

We examined whether baseline factors correlated with QuantiFERON-CMV results at Week 48 post-HCT (see Supplementary Results). Based on the findings from this analysis, known risk factors including conditioning regimen, stem cell source, donor type, and donor CMV serostatus had no meaningful impact on QuantiFERON-CMV results at Week 48 [1, 28].

In evaluating the predictive value of the assay, neither a positive nor a negative result was sufficiently discriminatory to provide added value in clinical decision-making compared with providing prophylaxis for a preset duration (based on the extended-duration prophylaxis study data [11]) without additional testing. Assay performance, evaluated using sensitivity, specificity, PPV, and NPV, was similar after completion of ∼100 or ∼200 days of letermovir prophylaxis. Although the PPV of the assay was high at both time points, NPV was consistently low. Furthermore, both the sensitivity and specificity of the assay were suboptimal at both time points. The totality of these data indicates that the utility of the QuantiFERON-CMV assay may be limited for predicting postprophylaxis CS-CMVi in the HCT setting.

Our findings are consistent with the limited clinical utility of the QuantiFERON-CMV assay observed in smaller observational studies [17, 29]. In a single-center, observational study of 35 adult recipients of allogeneic HCT, a positive QuantiFERON-CMV result was negatively correlated with CMV infection, though this was not statistically significant [29]. A prospective observational study of CMV-seropositive recipients of allogeneic HCT (n = 27; 4 of whom developed CS-CMVi) found that assay negativity at 100 days post-HCT was associated with the development of CS-CMVi at 100–270 days post-HCT [17]. The QuantiFERON-CMV assay showed high sensitivity (100%) and NPV (100%) for predicting CS-CMVi, but specificity (45.0%) and PPV (26.7%) were suboptimal.

The strength of this study, in addition to the large sample size, is the use of QuantiFERON-CMV testing on samples collected at prespecified time points in the setting of a global prospective clinical trial conducted at 32 centers in 6 countries, supporting the generalizability of our findings across diverse clinical settings. QuantiFERON-CMV results were not disclosed to investigators, ensuring that the outcome of interest was independently determined by investigators blinded to study treatment.

A potential limitation is that although all participants in the placebo group completed ∼100 days of letermovir prophylaxis (before entering the study), not all participants in the letermovir group completed ∼200 days of prophylaxis before the 200-day post-HCT CMV-CMI assessment [the median duration of prophylaxis after study entry in the letermovir group was 98 days {range, 39–104 days}].

A limitation of the QuantiFERON-CMV assay may be that it only detects IFN-y secreted by CD8+ T cells in response to specific CMV antigens [23]. When the extended-duration prophylaxis study was initiated, the QuantiFERON-CMV assay was the only commercially available assay for widespread implementation in a global study. Detection of IFN-y secreted by both CD4+ and CD8+ T cells may be necessary for a more complete assessment of CMV-CMI [26, 30–32], although more studies are needed to establish if including CD4+ T cells improves the reliability of IGRAs. The peptide-based enzyme-linked immunospot (ELISPOT) CMV assay measures CMV-specific IFN-y production by CD4+ and CD8+ T cells, and the CMV inSIGHT T-cell Immunity Panel quantifies the percentage of CMV-specific IFN-y–producing CD4+ and CD8+ T cells [16, 18–20, 33]. Additional limitations of the QuantiFERON-CMV assay include that it relies on human leukocyte antigen–restricted CMV epitopes to stimulate CD8+ T cells, and it often yields indeterminate results in immunocompromised patients [34]. Assay selection should consider the clinical utility in identifying protection against CS-CMVi, as well as feasibility, accessibility, and cost.

In conclusion, given the assay performance observed in these analyses of a prospective, global, multicenter, Phase 3 study, the utility of the QuantiFERON-CMV assay for predicting postprophylaxis CS-CMVi in routine clinical practice may be limited in the HCT setting. An unmet need remains for a standardized assay that can effectively distinguish between patients at risk of developing CS-CMVi and requiring antiviral prophylaxis from those who are protected.

## Supplementary Material

ciaf646_Supplementary_Data

## Supplementary Data

Supplementary materials are available at *Clinical Infectious Diseases* online. Consisting of data provided by the authors to benefit the reader, the posted materials are not copyedited and are the sole responsibility of the authors, so questions or comments should be addressed to the corresponding author.
