# Supplementary material for: An Analysis of Cytomegalovirus-Specific Cell-Mediated Immunity in a Phase 3, Randomized, Placebo-Controlled Trial of Letermovir Prophylaxis in Cytomegalovirus-Seropositive Recipients of an Allogeneic Hematopoietic Cell Transplant
Source: Clin Infect Dis. 2025 Dec 1;82(2):e308–15. doi: 10.1093/cid/ciaf646 (PMC13016986; doi:10.1093/cid/ciaf646)

SUPPLEMENTARY MATERIAL

An Analysis of Cytomegalovirus-Specific Cell-Mediated Immunity in a Phase 3, Randomized, Placebo-Controlled Trial of Letermovir Prophylaxis in Cytomegalovirus-Seropositive Recipients of an Allogeneic Hematopoietic Cell Transplant

Genovefa Papanicolaou* et al.

*Corresponding author (email: papanicg@mskcc.org)

Contents

SUPPLEMENTARY METHODS 2
  Definition of high-risk clinically significant cytomegalovirus infection 2
  Assessment of CMV-specific cell-mediated immunity using QuantiFERON-CMV assay.... 2
SUPPLEMENTARY RESULTS....... 3
  Impact of baseline factors on QuantiFERON-CMV results at Week 48 post-HCT....... 3
SUPPLEMENTARY TABLES....... 4
  Table S1. 4
  Table S2. 5
SUPPLEMENTARY FIGURES 6
  Figure S1. 6
  Figure S2. 7

SUPPLEMENTARY METHODS

Definition of high-risk clinically significant cytomegalovirus infection

The definition of participants at high risk of cytomegalovirus (CMV) infection and/or disease after completion of 100 days of letermovir prophylaxis included meeting ≥1 of the following criteria: (1) having a donor who was related with ≥1 mismatch at 1 of 3 specified HLA gene loci (HLA-A, HLA-B, or HLA-DR); (2) having a donor who was unrelated with ≥1 mismatch at 1 of 4 specified HLA gene loci (HLA-A, HLA-B, HLA-C, or HLA-DRB1); (3) receipt of hematopoietic stems cells from a donor who was haploidentical; (4) receipt of hematopoietic stem cells derived from umbilical cord blood; (5) receipt of ex vivo grafts depleted of T cells, antithymocyte globulin, or alemtuzumab; or (6) presence of graft-vs-host disease or other conditions necessitating use of systemic prednisone (or an equivalent) at a dose of ≥1 mg/kg per day within 6 weeks of randomization.

Assessment of CMV-specific cell-mediated immunity using QuantiFERON-CMV assay

The QuantiFERON-CMV (Qiagen, Inc; Hilden, Germany) assay utilizes a Mitogen positive control and a negative (Nil) control. Per the manufacturer’s cutoffs, a reactive QuantiFERON-CMV result (CMV minus Nil ≥0.20 IU/mL and significantly [≥25%] above the Nil control value) was interpreted as positive for CMV-specific cell-mediated immunity (CMV-CMI), and a nonreactive assay result (CMV minus Nil <0.20 IU/mL, or ≥0.20 IU/mL and <25% of the Nil control value) was interpreted as negative for CMV-CMI. An indeterminate assay result (Nil control value >8.0 IU/mL or Mitogen minus Nil value <0.5 IU/mL) was interpreted as indeterminate for CMV-CMI.

SUPPLEMENTARY RESULTS

Impact of baseline factors on QuantiFERON-CMV results at Week 48 post-HCT

To evaluate whether factors at baseline could meaningfully influence QuantiFERON-CMV results at Week 48 post-HCT, a univariate analysis using all variables reported in the demographics and baseline characteristics table (Table 1) was performed. Based on the univariate analysis, the following factors were included in a subsequent stepwise regression model: (1) race group (White vs other race), (2) conditioning regimen (myeloablative vs other), (3) stem cell source (bone marrow, cord blood, or peripheral blood), (4) donor type (matched related, matched unrelated, mismatched related, or mismatched unrelated), and (5) donor CMV serostatus (positive vs negative).

The following factors were then sequentially removed from the model as non-significant (using a 0.05 significance level for removal):

Summary of Backward Elimination

Step	Effect removed	Wald chi-square	p-value from the chi-square test
1	Conditioning regimen (myeloablative vs other)	0.1493	0.9281
2	Stem cell source	0.1414	0.7069
3	Donor type	2.2191	0.5282
4	Donor CMV serostatus	2.8470	0.0915

SUPPLEMENTARY TABLES

Table S1. Demographics and Baseline Characteristics of Participants in the Analysis Population for Evaluating the Clinical Utility of QuantiFERON-CMV Assay[a]

	**Letermovir (n=99)[b]**	**Placebo (n=58)[c]**	**Total (N=157)**
Sex, male, n (%)	67 (67.7)	33 (56.9)	100 (63.7)
Age, mean (SD), years	52.8 (13.4)	52.2 (12.6)	52.5 (13.1)
Race, n (%)			
Asian	13 (13.1)	8 (13.8)	21 (13.4)
Black or African American	1 (1.0)	0	1 (0.6)
Multiple	0 (0)	1 (1.7)	1 (0.6)
Native Hawaiian or other Pacific Islander	1 (1.0)	0	1 (0.6)
White	77 (77.8)	47 (81.0)	124 (79.0)
Not available[d]	7 (7.1)	2 (3.4)	9 (5.7)
Donor type, n (%)			
Matched related	9 (9.1)	11 (19.0)	20 (12.7)
Mismatched related	35 (35.4)	18 (31.0)	53 (33.8)
Matched unrelated	28 (28.3)	15 (25.9)	43 (27.4)
Mismatched unrelated	27 (27.3)	14 (24.1)	41 (26.1)
Stem cell source, n (%)			
Peripheral blood	83 (83.8)	49 (84.5)	132 (84.1)
Bone marrow	10 (10.1)	6 (10.3)	16 (10.2)
Cord blood	6 (6.1)	3 (5.2)	9 (5.7)
T-cell–depleting induction therapy, n (%)	56 (56.6)	36 (62.1)	92 (58.6)
Ex vivo T-cell–depleted grafts	5 (5.1)	6 (10.3)	11 (7.0)
Antithymocyte globulin	50 (50.5)	27 (46.6)	77 (49.0)
Alemtuzumab	6 (6.1)	9 (15.5)	15 (9.6)
Conditioning regimen use, n (%)			
Myeloablative	48 (48.5)	25 (43.1)	73 (46.5)
Reduced intensity conditioning	34 (34.3)	22 (37.9)	56 (35.7)
Nonmyeloablative	17 (17.2)	11 (19.0)	28 (17.8)

CMV, cytomegalovirus; HCT, hematopoietic cell transplant. [a]Includes all participants who had a positive or negative QuantiFERON-CMV result after completion of letermovir prophylaxis at 100 (placebo group) or 200 (letermovir group) days post-HCT. [b]Participants received ~100 days of letermovir prophylaxis post-HCT then completed extended-duration letermovir prophylaxis through 200 days post-HCT. [c]Participants completed ~100 days of letermovir prophylaxis post-HCT then received placebo through 200 days post-HCT. [d]Participants did not know their race or chose not to report due to local regulations.

Table S2. QuantiFERON-CMV Assay Performance for Predicting Post-Prophylaxis CS-CMVi Through Week 48 in the Analysis Population for Evaluating the Clinical Utility of QuantiFERON-CMV Assay With Pooled Indeterminate and Negative Results

**Assay performance for placebo group based on results after ~100 days of letermovir prophylaxis[a]**			
**QuantiFERON-CMV result**	**Did not develop post-prophylaxis CS-CMVi (n=58)**	**Developed post-prophylaxis CS-CMVi (n=14)**	**PPV/NPV, % (95% CI)**
Positive (n=29)	26 (TP)	3 (FP)	PPV, 89.7 (72.6, 97.8)
Negative + indeterminate (n=43)	32 (FN)	11 (TN)	NPV, 25.6 (13.5, 41.2)
Sensitivity/Specificity, % (95% CI)	Sensitivity, 44.8 (31.7, 58.5)	Specificity, 78.6 (49.2, 95.3)	—
**Assay performance for letermovir group based on results after ~200 days of letermovir prophylaxis[b]**			
**QuantiFERON-CMV result**	**Did not develop post-prophylaxis CS-CMVi (n=90)**	**Developed post-prophylaxis CS-CMVi (n=17)**	**PPV/NPV, % (95% CI)**
Positive (n=57)	51 (TP)	6 (FP)	PPV, 89.5 (78.5, 96.0)
Negative + indeterminate (n=50)	39 (FN)	11 (TN)	NPV, 22.0 (11.5, 36.0)
Sensitivity/Specificity, % (95% CI)	Sensitivity, 56.7 (45.8, 67.1)	Specificity, 64.7 (38.3, 85.8)	—

The 95% CIs were calculated based on exact binomial tests. Sensitivity = $\frac{TP}{TP+FN}$; Specificity = $\frac{TN}{TN+FP}$; PPV = $\frac{TP}{TP+FP}$; NPV = $\frac{TN}{TN+FN}$. CI, confidence interval; CMV, cytomegalovirus; CS-CMVi, clinically significant CMV infection; FN, false negative; FP, false positive; HCT, hematopoietic cell transplant; NPV, negative predictive value; PPV, positive predictive value; TN, true negative; TP, true positive. [a]Samples for QuantiFERON-CMV assay collected after completion of ~100 days of letermovir prophylaxis. Analysis population includes all placebo group participants who had positive, negative, or indeterminate QuantiFERON-CMV results at 100 days post-HCT. [b]Samples for QuantiFERON-CMV assay collected after completion of ~200 days of letermovir prophylaxis. Analysis population includes all letermovir group participants who had positive, negative, or indeterminate QuantiFERON-CMV results at 200 days post-HCT.

SUPPLEMENTARY FIGURES

Figure S1. Flow chart detailing the analysis population for assessing trends in QuantiFERON-CMV results. CMV, cytomegalovirus; HCT, hematopoietic cell transplant. [a]Participants received ~100 days of letermovir prophylaxis post-HCT then completed extended-duration letermovir prophylaxis through 200 days post-HCT. [b]Participants completed ~100 days of letermovir prophylaxis post-HCT then received placebo through 200 days post-HCT.

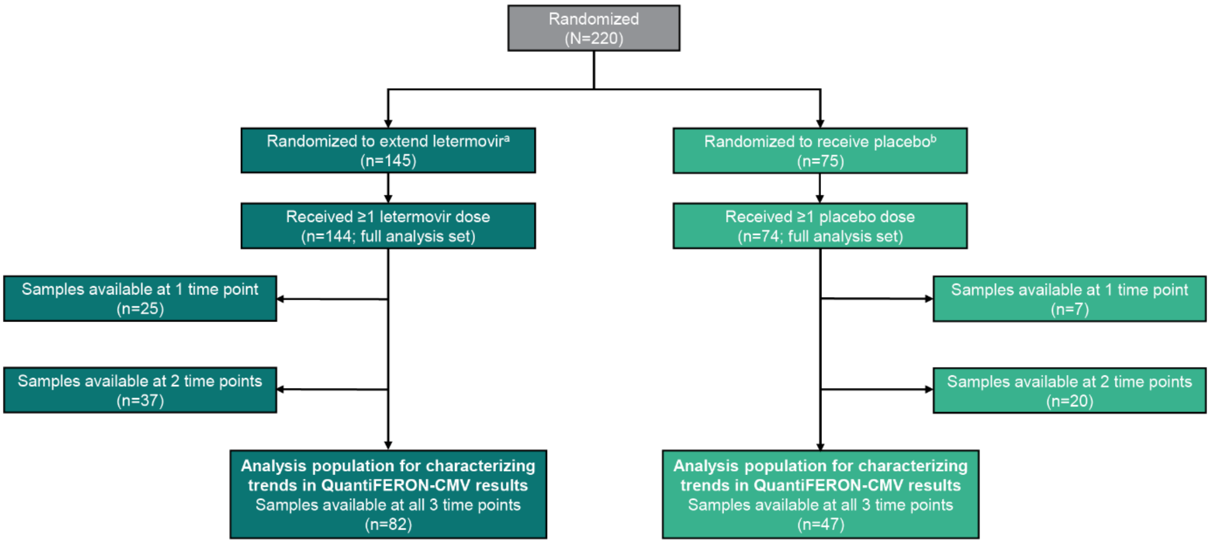

Figure S2. Flow chart detailing the analysis population for evaluating the clinical utility of QuantiFERON-CMV assay. CMV, cytomegalovirus; HCT, hematopoietic cell transplant. [a]Participants received ~100 days of letermovir prophylaxis post-HCT then completed extended-duration letermovir prophylaxis through 200 days post-HCT. [b]Participants completed ~100 days of letermovir prophylaxis post-HCT then received placebo through 200 days post-HCT.

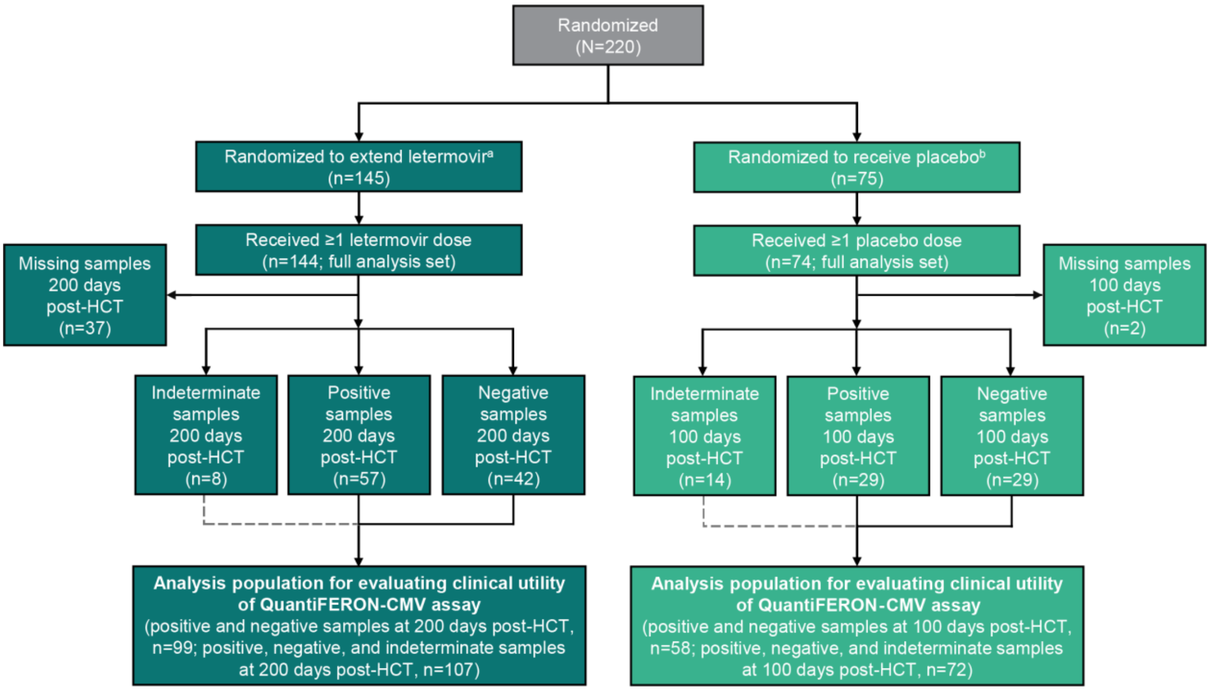

Supplement: ciaf646_Supplementary_Data [file ciaf646_supplementary_data.pdf]
